# MicroRNA related prognosis biomarkers from high throughput sequencing data of kidney renal clear cell carcinoma

**DOI:** 10.1186/s12920-021-00932-z

**Published:** 2021-03-09

**Authors:** Minjiang Huang, Ti Zhang, Zhi-Yong Yao, Chaoqung Xing, Qingyi Wu, Yuan-Wu Liu, Xiao-Liang Xing

**Affiliations:** 1 Hunan University of Medicine, Huaihua, 418000 Hunan People’s Republic of China; 2 The First Affiliated Hospital of Hunan University of Medicine, Huaihua, 418000 Hunan People’s Republic of China; 3 Beijing Advanced Innovation Center for Food Nutrition and Human Health, China Agricultural University, 100193 Beijing, People’s Republic of China

**Keywords:** KIRC, DEGs, DEMs, Overall survivals, Biomarkers

## Abstract

**Background:**

Kidney renal clear cell carcinoma (KIRC) is the most common type of kidney cell carcinoma which has the worst overall survival rate. Almost 30% of patients with localized cancers eventually develop to metastases despite of early surgical treatment carried out. MicroRNAs (miRNAs) play a critical role in human cancer initiation, progression, and prognosis. The aim of our study was to identify potential prognosis biomarkers to predict overall survival of KIRC.

**Methods:**

All data were downloaded from an open access database The Cancer Genome Atlas. DESeq2 package in R was used to screening the differential expression miRNAs (DEMs) and genes (DEGs). RegParallel and Survival packages in R was used to analysis their relationships with the KIRC patients. David version 6.8 and STRING version 11 were used to take the Gene Ontology and Kyoto Encyclopedia of Genes and Genomes pathway enrichment analysis.

**Results:**

We found 2 DEGs (TIMP3 and HMGCS1) and 3 DEMs (hsa-miR-21-5p, hsa-miR-223-3p, and hsa-miR-365a-3p) could be prognosis biomarkers for the prediction of KIRC patients. The constructed prognostic model based on those 2 DEGs could effectively predict the survival status of KIRC. And the constructed prognostic model based on those 3 DEMs could effectively predict the survival status of KIRC in 3-year and 5-year.

**Conclusion:**

The current study provided novel insights into the miRNA related mRNA network in KIRC and those 2 DEGs biomarkers and 3 DEMs biomarkers may be independent prognostic signatures in predicting the survival of KIRC patients.

**Supplementary Information:**

The online version contains supplementary material available at 10.1186/s12920-021-00932-z.

## Background

Kidney Cancer is one of the most common malignancies which account for 2.2% of all new cancer cases and 1.8% of all cancer related death in 2018 globally [1]. Renal cell carcinoma (RCC) accounts for 90% of all kidney cancers [2]. RCC could be divided into three categories: kidney renal clear cell carcinoma (KIRC), kidney renal papillary cell carcinoma (KIRP) and kidney chromophobe (KICG). Among them, KIRC is the most common type which accounts for about 70–75% of RCC [2]. Due to the radiotherapy and chemotherapy resistance, surgical treatment is currently the most effective way for KIRC patients [3]. However previous studies indicated that KIRC usually has the worst overall survival rate [2]. Almost 30% of patients with localized cancers eventually develop to metastases despite of early surgical treatment carried out [4]. Therefore, it is necessary to identify suitable prognosis biomarkers for the diagnosis and treatment of KIRC even though many prognosis factors have been reported for KIRC.

Cancer stem cells are a subpopulation of cells that has the driving force of carcinogenesis which is a complex and multistep phenomenon and involves accumulation of genetic and epigenetic changes ultimately leading to the development of pathological manifestations [5]. MicroRNAs (miRNAs) are a family of endogenous, small, noncodingRNAs. MiRNAs could regulate the expression of genes associated with various biological phenomenons such as homeostasis, development, proliferation, differentiation, and apoptosis [6, 7]. Deregulated expression and signaling of miRNA have been well-studied in the pathogenesis of various cancers. Aberrant expressions of miRNAs are vital for the initiation and progression of human malignancies as they act as both tumor suppressors and oncogenes [8]. In the present study, we aimed to identify potential prognosis biomarkers to predict overall survival of KIRC.

## Methods

### Data source and data processing

KIRC (71 controls vs 516 cancers) miRNA sequencing data, KIRC (72 controls vs 530 cancers) mRNA sequencing data and the corresponding clinical information were obtained from an open database TCGA. DESeq2 was used to identify the DEMs and DEGs according to |log2FC| > 0.5, basemean > 50, padj < 0.05. And we utilized two different web based tools, miRDB and TargetScanHuman 7.2, to screen the target genes of miRNAs. The target genes only enriched in two databases could be selected as putative target genes for the next analysis.

### Gene ontology (GO) and Kyoto encyclopedia of genes and genomes (KEGG) pathway enrichment analysis

DAVID version 6.8 was used to determine the association among target genes. To gain insight into the biological functions of those DEGs, GO and KEGG pathway enrichment analyses were performed.

### Protein–protein interaction (PPI) network and module analysis

STRING version 11 was used to assess PPI information. A normal medium confidence interval of 0.4 was used as threshold. Cytoscape_v3.7.2 software was used to visualize the resulting PPI network. The Molecular Complex Detection (MCODE) application was used to select significant modules from the PPI network in Cytoscape_v3.7.2.

### Pathologic TNM correlation analysis

After the classification base on the size and/or extent of the main cancer (T1 + 2 and T3 + 4), lymph node metastasis (N0 and N1), and distant metastasis (M0 and M1) respectively, we used Cox regression analysis, Kaplan–Meier curve, and log-rank analysis to further verify the characteristics of the pathologic TNM and the intensity of their correlation with survival. A repeated-measure ANOVA followed by Bonferroni post hoc tests or unpaired two-tail Student’s t test was used exam the correlation of DEGs with pathologic TNM.

### Survival analysis

Median is the number in the middle of a set of data in order. We divided the samples into high expression group and low expression group based on the median. We used RegParallel and survival packages in R to carry out univariate and multivariate Cox regression analysis.

### Specific prognostic model construction

After multivariate Cox regression analysis, we constructed specific prognostic models according to previous reports [9]. KIRC patients were divided into low risk group and high risk group depend on the median value of the risk score. Patients whose risk values were higher than the median were classified as high risk, and Patients whose risk values were lower than the median were classified as low-risk. And then we used survival analysis to know the relationship of the models with the survival of KIRC patient. And then we constructed time-dependent receiver operating characteristic (ROC) curves within 1-, 3-, and 5-year and estimated its utility as a prognostic model for predicting the survival status.

## Results

### Identification of DEMs and DEGs

**Fig. 1:**
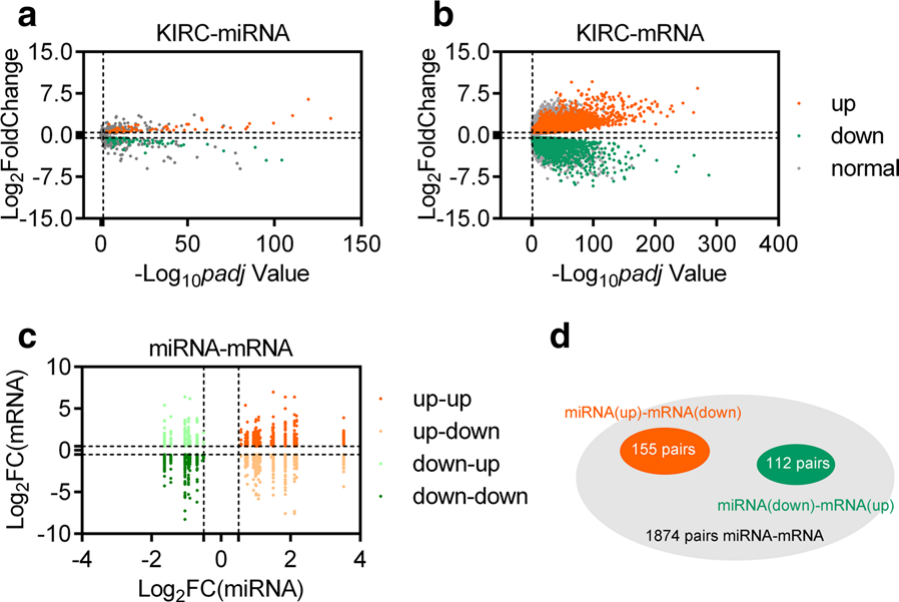
Differential miRNAs and mRNAs expression analysis.** a** Volcano plot of DEMs for KIRC. **b** Volcano plot of DEGs for KIRC. **c** Scatter plot of Log_2_FC(miRNA) versus Log_2_FC(mRNA) in KIRC. **d** The number of miRNAs-mRNAs verified by correlation analysis

TCGA is an open access database containing miRNA/mRNA profiles and the corresponding clinical information. Using Padj < 0.05 and |log2FC|> 0.5 as cut-off criteria for DEMs, 111 DEMs were identified by DESeq2 analysis, including 62 up-regulated DEMs and 59 down-regulated DEMs (Fig. 1a). Using basemean > 50, Padj < 0.05 and |log2FC| > 0.5 as cut-off criteria for DEGs, 8694 DEGs were identified by DESeq2 analysis, including 5288 up-regulated DEMs and 3406 down-regulated DEMs (Fig. 1b). Then we performed survival analysis for those 111 DEMs and found that 40 DEMs were correlated with the overall survival of KIRC patients. Of which 25 DEMs was up-regulated and 15 DEMs was down-regulated (Table 1).

**Table 1:** Candidate prognostic DEMs for KIRP by univariate Cox regression analysis

miRNA	HR	HRlower	HRupper	P.adj	LogRank.adj
hsa-miR-139-5p	0.407	0.295	0.563	0.000	0.000
hsa-miR-21-5p	2.327	1.700	3.186	0.000	0.000
hsa-miR-365a-3p	2.286	1.654	3.160	0.000	0.000
hsa-miR-365b-3p	2.286	1.654	3.160	0.000	0.000
hsa-miR-223-3p	2.205	1.612	3.017	0.000	0.000
hsa-miR-222-3p	2.053	1.506	2.799	0.000	0.000
hsa-miR-204-5p	0.493	0.361	0.674	0.000	0.000
hsa-miR-10b-3p	0.495	0.363	0.676	0.000	0.000
hsa-miR-221-3p	1.955	1.437	2.660	0.000	0.000
hsa-miR-126-3p	0.535	0.394	0.727	0.001	0.001
hsa-miR-10b-5p	0.542	0.399	0.736	0.001	0.001
hsa-miR-625-3p	1.866	1.368	2.545	0.001	0.001
hsa-miR-99b-5p	0.544	0.399	0.741	0.001	0.001
hsa-miR-146b-5p	1.819	1.338	2.473	0.001	0.001
hsa-miR-30c-2-3p	0.557	0.409	0.759	0.002	0.001
hsa-miR-101-3p	0.562	0.413	0.766	0.002	0.002
hsa-miR-130a-3p	1.759	1.294	2.391	0.002	0.002
hsa-miR-155-5p	1.773	1.300	2.419	0.002	0.002
hsa-miR-335-3p	1.685	1.243	2.284	0.005	0.004
hsa-miR-144-3p	0.594	0.437	0.808	0.005	0.005
hsa-miR-191-5p	1.683	1.236	2.292	0.005	0.005
hsa-miR-1269a	1.667	1.225	2.269	0.006	0.006
hsa-miR-30a-3p	0.615	0.454	0.833	0.008	0.008
hsa-miR-144-5p	0.612	0.451	0.831	0.008	0.008
hsa-miR-142-3p	1.610	1.187	2.184	0.010	0.009
hsa-miR-486-5p	0.619	0.455	0.841	0.010	0.009
hsa-let-7g-5p	1.593	1.173	2.164	0.012	0.011
hsa-miR-106b-5p	1.587	1.171	2.152	0.012	0.011
hsa-miR-451a	0.626	0.461	0.851	0.012	0.011
hsa-miR-146b-3p	1.548	1.143	2.096	0.019	0.018
hsa-let-7f-5p	0.652	0.481	0.885	0.022	0.021
hsa-let-7i-5p	1.534	1.131	2.079	0.022	0.021
hsa-miR-455-5p	0.651	0.479	0.884	0.022	0.021
hsa-miR-27b-3p	0.657	0.484	0.892	0.025	0.024
hsa-miR-21-3p	1.500	1.108	2.031	0.030	0.029
hsa-miR-584-5p	0.678	0.501	0.918	0.039	0.037
hsa-miR-2355-5p	0.678	0.502	0.917	0.039	0.037
hsa-miR-29c-5p	0.686	0.507	0.926	0.044	0.043
hsa-miR-192-3p	0.689	0.510	0.931	0.047	0.045
hsa-miR-342-3p	1.451	1.071	1.967	0.049	0.047

To ensure the integrity of the target genes, we utilized miRDB and TargetScanHuman7.2 for target gene prediction. Following integrated analysis of DEGs and target genes of DEMs, a total of 1874 pairs miRNAs-mRNAs were identified involved 1364 DEGs, including 647 up-regulated DEGs and 717 down-regulated DGEs (Fig. 1c). Since the relationship between DEGs and DEMs is negatively correlated, we introduced the spearman correlation analysis by using *p* < 0.05 and r < − 0.3 as cut-off criteria. And we obtained 267 pairs miRNAs-mRNAs which contained 20 DEMs (such as, Top 3 up-regulated DEMs: hsa-miR-155-5p, hsa-miR-21-5p, and hsa-miR-584-5p. Top 3 down-regulated DEMs: hsa-miR-204-5p, hsa-miR-30c-2-3p, and hsa-miR-30a-3p) and 252 DEGs (such as, Top 3 up-regulated DEMs: IGLON5, SOX11, and LOX. Top 3 down-regulated DEMs: EHF, MUC15, and CALB1) (Fig. 1d).

### GO function and KEGG pathway enrichment analysis of the DEGs.

**Fig. 2:**
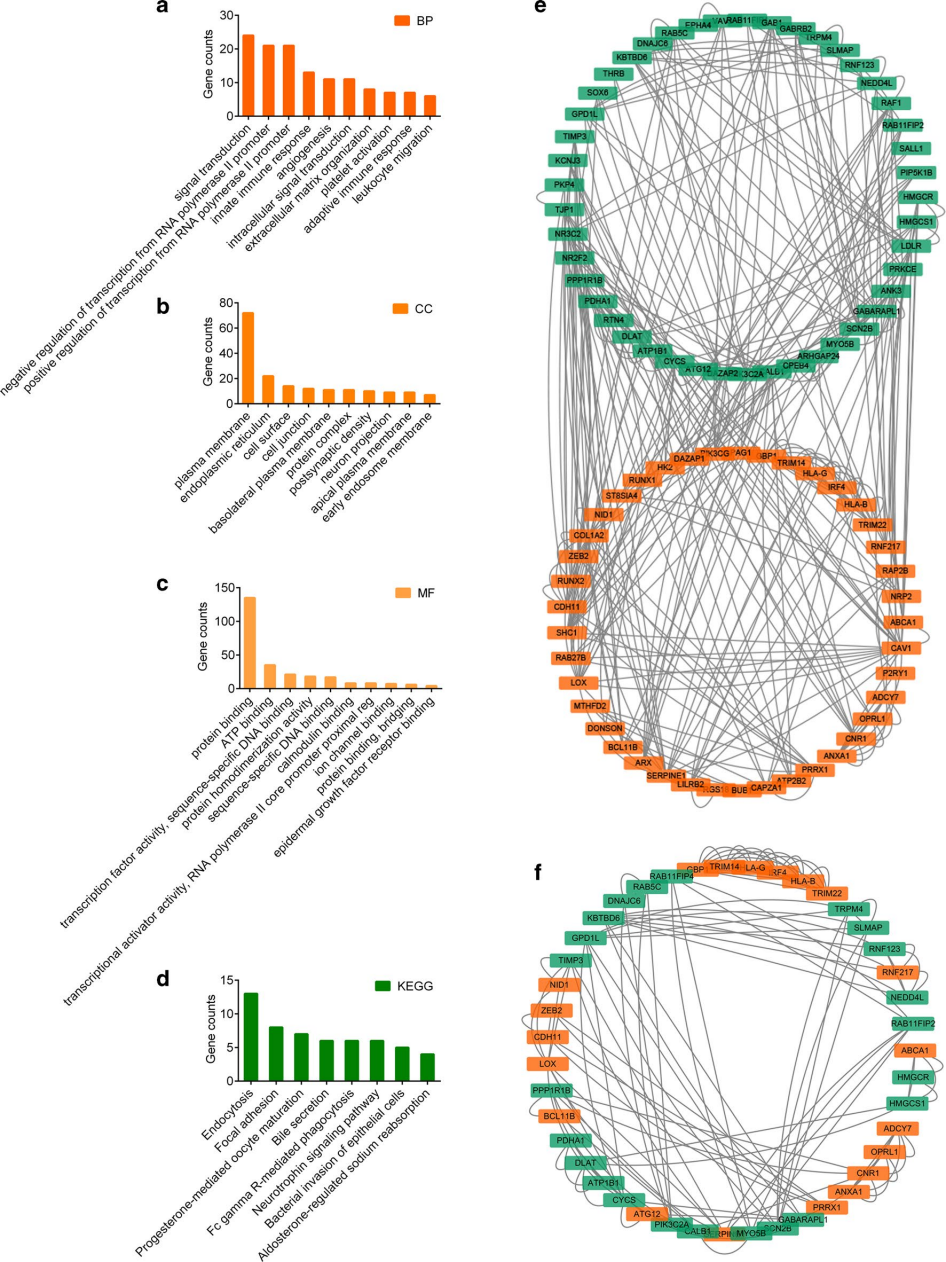
Functional enrichment analysis and PPI network construction. **a–c** The significantly enriched top 10 GO term (*p* value < 0.05) analyzed by David 6.8 for KIRC. **d** The significantly enriched KEGG pathway (*p* value < 0.05) analyzed by David 6.8 for KIRC. **e** PPI network of DEGs with their degree higher than the average in KIRC. **f** PPI network of DEGs verified by MCODE analysis. The orange represents un-regulated genes; the green represents down-regulated genes

To gain a deeper understanding of the selected DEGs, we performed GO and KEGG analysis for those 252 DEGs. There were 65 biological process (BP), 23 cellular component (CC), and 29 molecular functions (MF) that were enriched by GO analysis (such as, Top 3 GO-BP, signal transduction, negative regulation of transcription from RNA polymerase II promoter, and positive regulation of transcription from RNA polymerase II promoter. Top 3 GO-CC, plasma membrane, endoplasmic reticulum, and cell surface. Top 3 GO-MF, protein binding, ATP binding, and transcription factor activity, sequence-specific DNA binding) (Fig. 2a–c, Additional file 1: Table S1). And there were 20 KEGG pathways that were enriched by KEGG analysis, of which 9 signaling pathway was enriched significantly (such as Top 3 signaling pathway, hsa04144: Endocytosis, hsa04510: Focal adhesion, and hsa04914: Progesterone-mediated oocyte maturation) (Fig. 2d, Additional file 1: Table S2).

### PPI network construction and module selection.

Using the STRING database and Cytoscape software, a total of 252 DEGs were filtered into the PPI network, containing 252 nodes and 274 edges. According to the view that highly connected genes can have a major impact on disease, we identified 86 DEGs (with degree > average 2.17) as high connectivity genes in our study (Fig. 2e). According to their degree of importance, 10 important modules involved 43 DEGs from the PPI network complex were selected for further analysis based on Cytoscape MCODE (Fig. 2f). By cross analysis of those 86 DEGs and those 43 DEGs, we identified 10 overlap DEGs (ANXA1, BCL11B, CYCS, HLA-G, HMGCS1, RAB5C, RNF123, TIMP3, TRPM4, ZEB2).

### Pathologic TNM correlation analysis

**Fig. 3:**
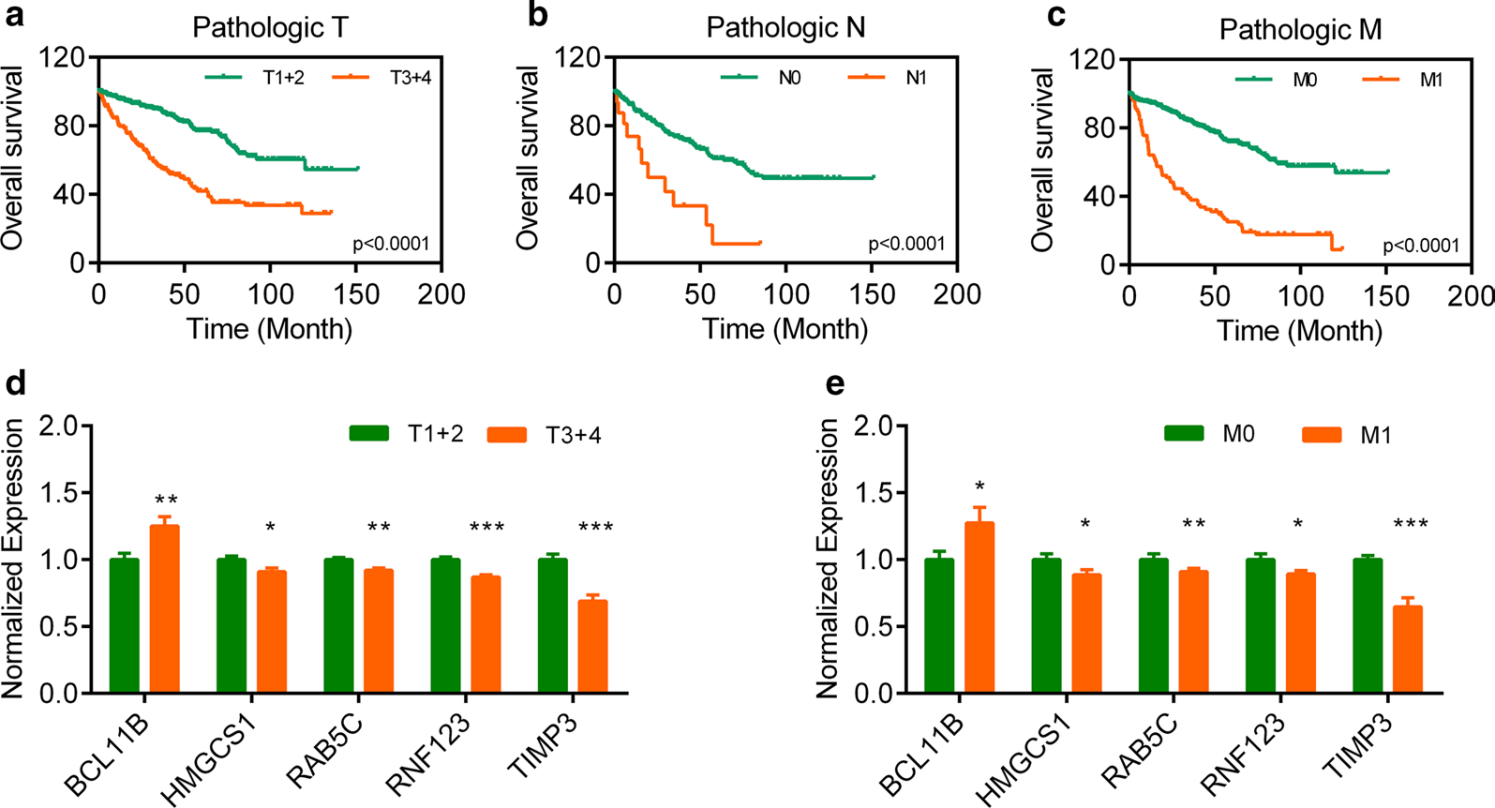
Pathologic TNM correlation analyses. **a**–**c** Survival curves of pathologic TNM for KIRC. **d** Associated analyses of DEGs with pathologic T stage for KIRC. **e** Associated analyses of DEGs with pathologic M stage for KIRC. *p < 0.05, **p < 0.01, ***p < 0.001

We classified the pathologic TNM staging of KIRC patients and conducted an overall survival correlation analysis. The results indicated that pathologic TNM were actually correlated with the overall survival. The KIRC patients with bigger of the size and/or extent of the main cancer (T3 + T4), or lymph node metastasis (N1), or distant metastasis (M1) displayed the worse overall survival rate (Fig. 3a–c). Subsequently, we evaluated their relationship of those 10 overlap DEGs with pathologic TNM, and found that 5 DEGs was confirmed to be associated with pathologic T and pathologic M (Fig. 3d–e).

### Specific prognostic model construction

**Fig. 4:**
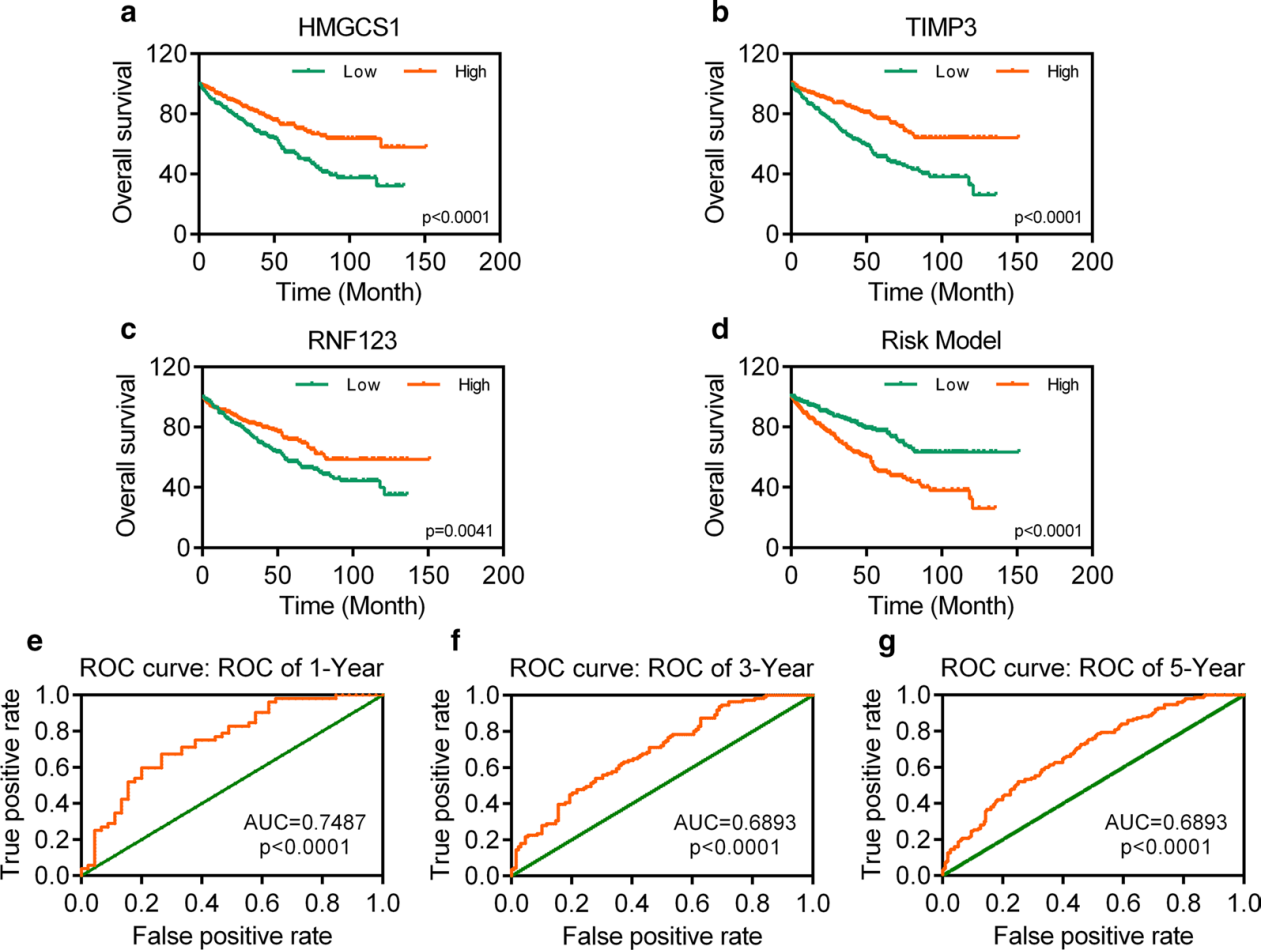
Construction of survival risk score system based on DEGs signature.** a–c** Survival curves of DEGs in KIRC. **d** Survival curves of prognostic model based on DEGs in KIRC. **e–g** The ROC curve of prognostic model based on DEGs in 1-, 3-, and 5-year with AUC value

After pathologic TNM correlation analysis, we performed univariate Cox regression analysis for those 10 DEGs. We found that HMGCS1, TIMP3, and RNF123 were correlated with overall survival. The KIRC patients with high expression of HMGCS1, TIMP3, and RNF123 exhibited a better overall survival (Fig. 4a–c). Then, we performed multivariate Cox regression analysis for HMGCS1, TIMP3, and RNF123. And the result showed that TIMP3 (*p* = 0.0001) and HMGCS1 (*p* = 0.0110) were still correlated with the overall survival of KIRC patients, of which the expression of TIMP3 (logFC = − 0.766, *p* < 0.001) and HMGCS1 (logFC = − 0.643, *p* < 0.001) were decreased significantly.

Followed the multivariate Cox regression analysis, we constructed a prognostic model by using TIMP3 and HMGCS1. The patients with low risk actually exhibited a better overall survival (Fig. 4d). The time-dependent receiver operating characteristic (ROC) curves had area under curve (AUC) values higher than 0.5, which were 0.7487, 0.6893, and 0.6893 respectively (Fig. 4e–g).

**Fig. 5:**
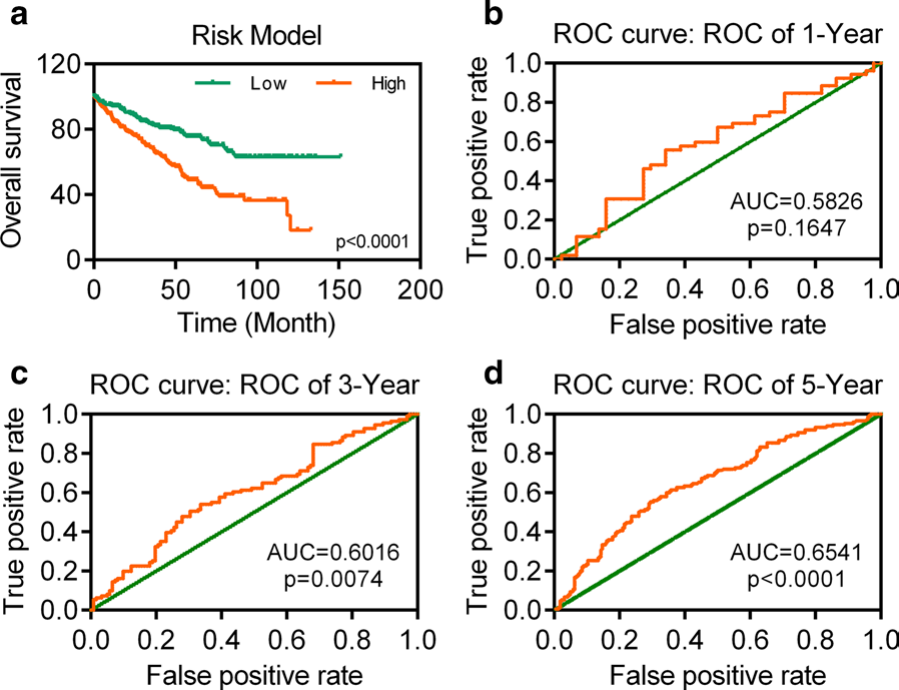
Construction of survival risk score system based on DEMs signature.** a** Survival curves of prognostic model based on DEMs in KIRC. **b**–**d** The ROC curve of prognostic model based on DEMs in 1-, 3-, and 5-year with AUC value

We screen the negative related miRNAs of TIMP3 and HMGCS1 among those 20 DEMs, and found TIMP3 was correlated with hsa-miR-21-5p (r = − 0.520), HMGCS1 were correlated with hsa-miR-223-3p (r = − 0.318) and hsa-miR-365a-3p (r = − 0.340). The result of multivariate Cox regression analysis showed that hsa-miR-21-5p [*p* = 0.0002, HR = 1.854(1.343–2.560)], hsa-miR-223-3p [*p* = 0.0002, HR = 1.821(1.324–2.506)], and hsa-miR-365a-3p [*p* = 0.0004, HR = 1.823(1.309–2.538)] were still correlated with the overall survival of KIRC. And the expression of hsa-miR-21-5p (logFC = 2.167, *p* < 0.001), hsa-miR-223-3p (logFC = 1.000, *p* < 0.001), and hsa-miR-365a-3p (logFC = 0.998, *p* < 0.001) were increased significantly. The patient with low risk score actually exhibited a better overall survival (Fig. 5a). The AUC of the DEMs signature in 1-year was 0.5826 (Fig. 5b). And the time-dependent ROC curves in 3-year and 5-year had AUC values higher than 0.6, which were 0.6016 and 0.6541 respectively (Fig. 5c, d).

## Discussion

Previous study indicated that 2.2% of all new cancer cases and 1.8% of all cancer related death was RCC in 2018 globally [1]. And almost 30% of KIRC, the main type of RCC, with localized cancers eventually develop to metastases despite after surgical treatment [4]. Therefore, it is necessary to identify DEGs as suitable prognosis biomarkers for the diagnosis of KIRC. The expression of mRNA was affected by many factors, such as miRNA, lncRNA, methylation, and so on. In the present study, we just focus on the miRNAs. MiRNAs, a class of small noncoding RNAs of ∼22nt in length, are firstly identified in 1993 in *Caenorhabditis eleganss*. Increasing scientific reports demonstrate that miRNAs are involved in the regulation of almost all the biological phenomena in various species which repress the target transcripts through partial complementarity [5]. Abnormal expression of miRNAs is closely related to the pathogenesis of most human diseases, including cancer [8, 10]. Previous studies have shown that miRNAs are involved in the initiation, progression, and prognosis of various cancers. For instance, Zhang et al. found that miR-1246 and miR-1290 are critical for the tumor initiation and progression of lung cancer [11]. Wang et al. found that miR-200c targets CDK2 and suppresses tumorigenesis in renal cell carcinoma [12]. Therefore, the aim of this study was to find the biomarkers related correlated to abnormal miRNA expression, which has an important role in the diagnosis and prognosis for various cancers. In the present study, we found 20 DEMs and 252 DEGs through correlation analysis. And then, we found 20 KEGG pathways were enriched by functional enrichment analysis, of which 9 signaling pathway was enriched significantly. The other 11 signaling pathways are not significantly enriched, but previous studies also indicated that they also play an important role in the pathogenesis of cancers, such as metabolic related pathways [13–15].

Subsequently, we identified that TIMP3 and HMGCS1 were correlated with the overall survival of KIRC patients through bioinformatics analysis. And the constructed prognosis model based on TIMP3 and HMGCS1 could accurately predict the overall survival rate of KIRC patients. TIMP3 (Tissue inhibitor of metalloproteinases-3) belongs to a family of negative regulators of matrix metalloproteinase activity. Previous studies indicated that TIMP3 as a tumor suppressor could modulate tumor migration, invasion, and tumorigenicity [16]. High expression of TIMP3 could promote apoptosis in various tumors [16]. Das et al. found that reduced expression of TIMP3 was observed in 74% of the human malignant melanoma cases [17]. Loss or down regulation of TIMP3 could promote the metastasis, cell growth and invasion of several cancers [18–20]. Moreover, Gu et al. and Mylona et al. found that TIMP3 could predict the overall survival rate for hepatocellular carcinoma and breast cancer [21, 22]. In the present study, we also found that the expression of TIMP3 was decreased significantly in KIRC. And the survival analysis also indicated that TIMP3 was correlated with the overall survival rate of KIRC patients which further reinforce the relationship of TIMP with cancers. The KIRC patients with low expression of TIMP3 displayed worse overall survival rate. All of these results reinforced the relationship of TIMP3 with cancers. By retrospective analysis, we found hsa-miR-21-5p was also correlated with overall survival which could be a potential prognosis biomarker for KIRC. Park et al. found hsa-miR-21-5p was more highly expressed in the recurrence group than in the nonrecurrence group of gastric cancer [23]. Chang et al. found that hsa-miR-21-5p may exert protective phenotypes by targeting breast oncogenes that contribute to patient survival [24].

HMGCS1 (3-hydroxy-3-methylglutaryl-CoA synthase 1) is a potential regulatory node in the mevalonate pathway, whose up-regulation is a common transcriptional event in cancer stem cell enriched subpopulations of breast cancer cell lines [25]. Wang et al. also found the expression of HMGCS1 is increased significantly in stomach adenocarcinoma samples of patients and tumorspheres of gastric cancer cells [26]. Additionally, previous reports demonstrated that krüppel-like factors (KLFs) could regulate the expression of various substrates. Yao et al. found that KLF13 was downregulated in colorectal cancer tissues and colorectal cancer cell line [27]. KLF13 knockdown could effectively promote cell proliferation and colony formation. Opposite results were observed in KLF13 overexpressed cells [27]. In the further research, Yao et al. found that KLF13 transcriptionally inhibited HMGCS1 and knockdown of HMGCS1 could suppress the proliferation of colorectal cancer [27]. But in the present study, we found that the expression of HMGCS1 was decreased significantly in KIRC. And by retrospective analysis, we found HMGCS1 correlated miRNAs hsa-miR-223-3p and hsa-miR-365a-3p were correlated with the overall survival of KIRC. The expression of hsa-miR-223-3p and hsa-miR-365a-3p were increased significantly in KIRC. Previous studies indicated that hsa-miR-223-3p and hsa-miR-365a-3p could promote proliferation, migration and invasion of cancer cells [28–31]. But what very interesting is that previous studies also indicated that hsa-miR-223-3p and hsa-miR-365a-3p could inhibit the invasion and migration of cancer cells [32–35]. All of those results suggested that the same gene or miRNA may play different roles in different cancers.

## Conclusion

In the present study, we found 2 DEGs and 3 DEMs could be the candidate prognosis biomarkers for KIRC patients. The constructed risk models based on 2 DEGs and 3 DEMs could accurately predict the outcome. We just provided an analysis direction depended on theoretical knowledge and clinical outcomes, more scientific research, especially clinical studies, were needed to confirm our findings.

## Supplementary Information

**Additional file 1: Supplementary Table S1.** GO function enrichment analysis of the DEGs. **Supplementary Table 2**. KEGG pathway enrichment analysis of the DEGs.

## Data Availability

The datasets analysed during the current study are available in TCGA repository (https://portal.gdc.cancer.gov/projects/TCGA-KIRC), under the accession code: Kidney Renal Clear Cell Carcinoma (KIRC).

## Abbreviations

KIRC: Kidney renal clear cell carcinoma
DEGs: Differential expression genes
DEMs: Differential expression miRNAs
