# Supplementary material for: MicroRNA related prognosis biomarkers from high throughput sequencing data of kidney renal clear cell carcinoma
Source: BMC Med Genomics. 2021 Mar 9;14:72. doi: 10.1186/s12920-021-00932-z (PMC7941961; doi:10.1186/s12920-021-00932-z)
Supplement: Supplementary file 1 — Additional file 1: Supplementary Table S1. GO function enrichment analysis of the DEGs. Supplementary Table 2. KEGG pathway enrichment analysis of the DEGs. [file 12920_2021_932_MOESM1_ESM.docx]

**MicroRNA related prognosis biomarkers from high throughput sequencing data of kidney renal clear cell carcinoma**

Minjiang Huang^1#^, Ti Zhang^1#^, Zhi-Yong Yao^1^, Chaoqung Xing^2^, Qingyi Wu^1^, Yuan-Wu Liu^3^, Xiao-Liang Xing^1*^

^1^Hunan University of Medicine, Huaihua 418000, Hunan, P. R. China.

^2^The first Affiliated Hospital of Hunan University of Medicine, Huaihua 418000, Hunan, P. R. China.

^3^Beijing Advanced Innovation Center for Food Nutrition and Human Health, China Agricultural University, 100193, Beijing, China.

^#^Contributed equally to this work

*Correspondence: Xiao-Liang Xing, xiaoliangxinghnm@126.com

**Supplementary table: 2 tables**

**Supplementary table 1 GO function enrichment analysis of the DEGs**

| Term | | Count | PValue | Genes |
| --- | --- | --- | --- | --- |
| BP | GO:0007605~sensory perception of sound | 6 | 0.033 | TJP1, GABRB2, PGAP1, THRB, SLC1A3, ATP2B2 |
|  | GO:0060333~interferon-gamma-mediated signaling pathway | 5 | 0.015 | IRF4, HLA-B, GBP1, HLA-G, TRIM22 |
|  | GO:0007584~response to nutrient | 5 | 0.017 | ABCA1, CNR1, ACSL4, HMGCR, C2 |
|  | GO:0001503~ossification | 5 | 0.022 | IGSF10, CDH11, SUCO, RUNX2, RUNX1 |
|  | GO:0046854~phosphatidylinositol phosphorylation | 5 | 0.038 | GAB1, PIP4K2A, PIP5K1B, PIK3C2A, PIK3CG |
|  | GO:0086091~regulation of heart rate by cardiac conduction | 4 | 0.011 | CAV1, KCNJ3, SCN2B, TRPM4 |
|  | GO:0030199~collagen fibril organization | 4 | 0.015 | COL1A2, LOX, COL5A3, CYP1B1 |
|  | GO:0071230~cellular response to amino acid stimulus | 4 | 0.025 | COL1A2, SESN3, CPEB3, CPEB4 |
|  | GO:0007173~epidermal growth factor receptor signaling pathway | 4 | 0.039 | SHC1, GAB1, PIK3C2A, PAG1 |
|  | GO:0043401~steroid hormone mediated signaling pathway | 4 | 0.041 | NR6A1, THRB, NR2F2, NR3C2 |
|  | GO:0006661~phosphatidylinositol biosynthetic process | 4 | 0.043 | PIP4K2A, PIP5K1B, PIK3C2A, PIK3CG |
|  | GO:0009967~positive regulation of signal transduction | 4 | 0.048 | VAV3, HOMER1, SH2B3, PAG1 |
|  | GO:0001893~maternal placenta development | 3 | 0.007 | DAZAP1, NR2F2, PRDM1 |
|  | GO:0008347~glial cell migration | 3 | 0.007 | EPHA4, P2RY1, MATN2 |
|  | GO:0010765~positive regulation of sodium ion transport | 3 | 0.026 | WNK3, ANK3, GPD1L |
|  | GO:0030879~mammary gland development | 3 | 0.032 | HOXB9, CAV1, TBX3 |
|  | GO:0043278~response to morphine | 3 | 0.038 | CNR1, PRKCE, ADA |
|  | GO:0051928~positive regulation of calcium ion transport | 3 | 0.047 | HOMER1, WNK3, ATP2B2 |
|  | GO:2001198~regulation of dendritic cell differentiation | 2 | 0.027 | HLA-B, LILRB2 |
|  | GO:0002645~positive regulation of tolerance induction | 2 | 0.027 | LILRB2, HLA-G |
|  | GO:0002767~immune response-inhibiting cell surface receptor signaling pathway | 2 | 0.040 | LILRB2, HLA-G |
|  | GO:0030104~water homeostasis | 2 | 0.040 | AQP9, NEDD4L |
|  | GO:0043368~positive T cell selection | 2 | 0.040 | BCL11B, THEMIS |
|  | GO:0021957~corticospinal tract morphogenesis | 2 | 0.040 | EPHA4, CDH11 |
|  | GO:0072659~protein localization to plasma membrane | 4 | 0.052 | SLMAP, P2RY1, ANK3, ATP1B1 |
|  | GO:0002667~regulation of T cell anergy | 2 | 0.053 | PHLPP1, HLA-B |
|  | GO:0007621~negative regulation of female receptivity | 2 | 0.053 | THRB, PPP1R1B |
|  | GO:0002666~positive regulation of T cell tolerance induction | 2 | 0.053 | LILRB2, HLA-G |
|  | GO:0030242~pexophagy | 2 | 0.053 | ACBD5, TRAPPC8 |
|  | GO:0034220~ion transmembrane transport | 7 | 0.064 | GABRB2, TTYH1, NEDD4L, ATP2B2, ATP1B1, RAF1, ATP6V1E2 |
|  | GO:0072540~T-helper 17 cell lineage commitment | 2 | 0.065 | IRF4, SLAMF6 |
|  | GO:0003091~renal water homeostasis | 3 | 0.068 | MYO5B, RAB11FIP2, ADCY7 |
|  | GO:0051480~regulation of cytosolic calcium ion concentration | 3 | 0.068 | CALB1, CAV1, ATP2B2 |
|  | GO:0048167~regulation of synaptic plasticity | 3 | 0.068 | CALB1, ATP2B2, CPEB3 |
|  | GO:0002931~response to ischemia | 3 | 0.072 | CAV1, HK2, CPEB4 |
|  | GO:2000651~positive regulation of sodium ion transmembrane transporter activity | 2 | 0.078 | WNK3, ANK3 |
|  | GO:0046632~alpha-beta T cell differentiation | 2 | 0.078 | ANXA1, BCL11B |
|  | GO:0035773~insulin secretion involved in cellular response to glucose stimulus | 2 | 0.078 | RAF1, RAB11FIP2 |
|  | GO:0090279~regulation of calcium ion import | 2 | 0.078 | HOMER1, WNK3 |
|  | GO:0045591~positive regulation of regulatory T cell differentiation | 2 | 0.078 | LILRB2, HLA-G |
|  | GO:0002686~negative regulation of leukocyte migration | 2 | 0.078 | HOXA7, ADA |
|  | GO:0009396~folic acid-containing compound biosynthetic process | 2 | 0.078 | MTHFD1L, MTHFD2 |
|  | GO:0008286~insulin receptor signaling pathway | 4 | 0.087 | SHC1, GAB1, PIK3C2A, ATP6V1E2 |
|  | GO:0045893~positive regulation of transcription, DNA-templated | 12 | 0.088 | EHF, SALL1, IRF4, HIVEP3, SOX11, NR2F2, CRLF3, ARNTL2, RUNX2, TBX3, NPAS3, RUNX1 |
|  | GO:0042130~negative regulation of T cell proliferation | 3 | 0.088 | NDFIP1, LILRB2, HLA-G |
|  | GO:0032467~positive regulation of cytokinesis | 3 | 0.088 | KIF3B, PRKCE, PKP4 |
|  | GO:0007588~excretion | 3 | 0.088 | NFAT5, AQP9, NEDD4L |
|  | GO:2000766~negative regulation of cytoplasmic translation | 2 | 0.090 | CPEB3, CPEB4 |
|  | GO:0006086~acetyl-CoA biosynthetic process from pyruvate | 2 | 0.090 | PDHA1, DLAT |
|  | GO:0009649~entrainment of circadian clock | 2 | 0.090 | PHLPP1, ARNTL2 |
|  | GO:0007127~meiosis I | 2 | 0.090 | M1AP, SCIMP |
|  | GO:0006811~ion transport | 5 | 0.091 | GABRB2, SLC22A15, SLC1A3, LRRC55, TOMM40L |
|  | GO:0050776~regulation of immune response | 6 | 0.092 | COL1A2, HLA-B, SLAMF6, LILRB2, CLEC2D, HLA-G |
|  | GO:0010811~positive regulation of cell-substrate adhesion | 3 | 0.092 | PRKCE, ITGA5, NID1 |
|  | GO:0043085~positive regulation of catalytic activity | 4 | 0.095 | DBF4, SH3PXD2A, CAV1, PIK3CG |
| CC | GO:0045202~synapse | 7 | 0.033 | GABRB2, CALB1, DNAJC6, ATP2B2, ITGA5, CPEB3, CPEB4 |
|  | GO:0030670~phagocytic vesicle membrane | 5 | 0.008 | RAB31, HLA-B, TLR6, HLA-G, ATG12 |
|  | GO:0005811~lipid particle | 4 | 0.056 | RAB5C, CAV1, FAAH2, ACSL4 |
|  | GO:0043025~neuronal cell body | 9 | 0.058 | MYO1D, CALB1, MYO10, PPP1R1B, SLC1A3, ACSL4, ATP2B2, ADA, TRPM4 |
|  | GO:0045211~postsynaptic membrane | 7 | 0.062 | GABRB2, EPHA4, HOMER1, P2RY1, ANK3, CPEB3, CPEB4 |
|  | GO:0030424~axon | 7 | 0.074 | MYO1D, EPHA4, FAM168B, NRP2, CALB1, HOMER1, CNR1 |
|  | GO:0045177~apical part of cell | 4 | 0.076 | TJP1, HOMER1, CTSV, LDLR |
|  | GO:0045254~pyruvate dehydrogenase complex | 2 | 0.076 | PDHA1, DLAT |
|  | GO:1990124~messenger ribonucleoprotein complex | 2 | 0.076 | CPEB3, CPEB4 |
|  | GO:0043235~receptor complex | 5 | 0.087 | FCRL5, NT5DC3, LIFR, LDLR, NR3C2 |
|  | GO:0045179~apical cortex | 2 | 0.089 | GM2A, MYO5B |
|  | GO:0005829~cytosol | 53 | 0.095 | GABRB2, WWC1, PIK3C2A, HK2, PIK3CG, RPS6KA6, CALB1, PIP4K2A, ATP6V1E2, TRIM22, EDARADD, VAV3, GABARAPL1, HMGCS1, PRKCE, GAB1, ANK3, ATG12, RAP2B, MTHFD2, IRF4, DNAJC6, MAP1B, ARHGEF3, RAF1, PHLPP1, SHC1, NEDD4L, NOD1, ADD3, KIF3B, MTHFD1L, MKNK1, RNF217, NT5DC3, PIP5K1B, SH2B3, BUB1, GBP1, MAPK4, TRPM4, MYO10, ARHGAP24, MYO1D, TJP1, RGP1, ETNK2, CAPZA1, PPP1R1B, WNK3, CYCS, GPD1L, ADA |
|  | GO:0016020~membrane | 37 | 0.099 | NRP2, STAU2, SLC1A3, TFCP2L1, SLC7A11, PIK3C2A, ADD3, HK2, PIK3CG, SNX4, KIF3B, MTHFD1L, NIPAL1, PIP5K1B, PLXNC1, LDLR, BUB1, ACBD5, PPP1R21, CAV1, GALNT1, HLA-B, SUCO, ACSL4, LILRB2, ATP1B1, SCIMP, HLA-G, RGP1, RAP2B, IRF4, CDH11, TLR10, ASPHD2, CLEC2D, PRR11, ADA |
| MF | GO:0019003~GDP binding | 4 | 0.037 | RAB5C, RAB31, RAP2B, RAB27B |
|  | GO:0003707~steroid hormone receptor activity | 4 | 0.040 | NR6A1, THRB, NR2F2, NR3C2 |
|  | GO:0046875~ephrin receptor binding | 3 | 0.048 | EPHA4, SHC1, PIK3CG |
|  | GO:0003785~actin monomer binding | 3 | 0.048 | COBLL1, PRKCE, PFN2 |
|  | GO:0030507~spectrin binding | 3 | 0.048 | MYO10, ANK3, GBP1 |
|  | GO:0004488~methylenetetrahydrofolate dehydrogenase (NADP+) activity | 2 | 0.053 | MTHFD1L, MTHFD2 |
|  | GO:0004329~formate-tetrahydrofolate ligase activity | 2 | 0.053 | MTHFD1L, MTHFD2 |
|  | GO:0004477~methenyltetrahydrofolate cyclohydrolase activity | 2 | 0.053 | MTHFD1L, MTHFD2 |
|  | GO:0000978~RNA polymerase II core promoter proximal region sequence-specific DNA binding | 10 | 0.053 | NFAT5, EHF, NR6A1, SALL1, BCL11B, IRF4, PRDM1, HOXA7, RUNX2, TBX3 |
|  | GO:0008135~translation factor activity, RNA binding | 3 | 0.059 | SOX11, CPEB3, CPEB4 |
|  | GO:0052811~1-phosphatidylinositol-3-phosphate 4-kinase activity | 2 | 0.066 | PIP4K2A, PIP5K1B |
|  | GO:0035004~phosphatidylinositol 3-kinase activity | 2 | 0.066 | PIK3C2A, PIK3CG |
|  | GO:0017080~sodium channel regulator activity | 3 | 0.070 | NEDD4L, GPD1L, SCN2B |
|  | GO:0003828~alpha-N-acetylneuraminate alpha-2,8-sialyltransferase activity | 2 | 0.079 | ST8SIA1, ST8SIA4 |
|  | GO:0015288~porin activity | 2 | 0.079 | AQP9, TOMM40L |
|  | GO:0051117~ATPase binding | 4 | 0.079 | ABCA1, SLN, CAV1, ATP1B1 |
|  | GO:0016307~phosphatidylinositol phosphate kinase activity | 2 | 0.091 | PIP4K2A, PIP5K1B |
|  | GO:0035005~1-phosphatidylinositol-4-phosphate 3-kinase activity | 2 | 0.091 | PIK3C2A, PIK3CG |
|  | GO:0016308~1-phosphatidylinositol-4-phosphate 5-kinase activity | 2 | 0.091 | PIP4K2A, PIP5K1B |

**Supplementary table 2 KEGG pathway enrichment analysis of the DEGs**

| Term | Count | PValue | Genes |
| --- | --- | --- | --- |
| hsa04972:Pancreatic secretion | 5 | 0.053 | RAB27B, ATP2B2, ATP1B1, ADCY7, SLC4A4 |
| hsa05416:Viral myocarditis | 4 | 0.056 | CAV1, HLA-B, CYCS, HLA-G |
| hsa04066:HIF-1 signaling pathway | 5 | 0.059 | PDHA1, MKNK1, SERPINE1, HK2, PIK3CG |
| hsa04915:Estrogen signaling pathway | 5 | 0.064 | SHC1, RAF1, ADCY7, PIK3CG, KCNJ3 |
| hsa05230:Central carbon metabolism in cancer | 4 | 0.074 | PDHA1, RAF1, HK2, PIK3CG |
| hsa04145:Phagosome | 6 | 0.079 | RAB5C, HLA-B, TLR6, ITGA5, ATP6V1E2, HLA-G |
| hsa04024:cAMP signaling pathway | 7 | 0.082 | VAV3, PPP1R1B, ATP2B2, ATP1B1, RAF1, ADCY7, PIK3CG |
| hsa05205:Proteoglycans in cancer | 7 | 0.085 | CAV1, GAB1, TIMP3, ANK3, ITGA5, RAF1, PIK3CG |
| hsa04114:Oocyte meiosis | 5 | 0.089 | RPS6KA6, CPEB3, ADCY7, BUB1, CPEB4 |
| hsa00562:Inositol phosphate metabolism | 4 | 0.094 | PIP4K2A, PIP5K1B, PIK3C2A, PIK3CG |
| hsa05220:Chronic myeloid leukemia | 4 | 0.097 | SHC1, RAF1, PIK3CG, RUNX1 |
| hsa04919:Thyroid hormone signaling pathway | 5 | 0.098 | SLCO1C1, THRB, ATP1B1, RAF1, PIK3CG |
